# Selenoprotein S Attenuates Tumor Necrosis Factor-*α*-Induced Dysfunction in Endothelial Cells

**DOI:** 10.1155/2018/1625414

**Published:** 2018-04-01

**Authors:** Siyuan Cui, Lili Men, Yu Li, Yingshuo Zhong, Shanshan Yu, Fang Li, Jianling Du

**Affiliations:** ^1^Department of Endocrinology, The First Affiliated Hospital of Dalian Medical University, Dalian, Liaoning 116011, China; ^2^Department of Immunology, Dalian Medical University, Dalian, Liaoning 116044, China

## Abstract

Endothelial dysfunction, partly induced by inflammatory mediators, is known to initiate and promote several cardiovascular diseases. Selenoprotein S (SelS) has been identified in endothelial cells and is associated with inflammation; however, its function in inflammation-induced endothelial dysfunction has not been described. We first demonstrated that the upregulation of SelS enhances the levels of nitric oxide and endothelial nitric oxide synthase in tumor necrosis factor- (TNF-) *α*-treated human umbilical vein endothelial cells (HUVECs). The levels of TNF-*α*-induced endothelin-1 and reactive oxygen species are also reduced by the upregulation of SelS. Furthermore, SelS overexpression blocks the TNF-*α*-induced adhesion of THP-1 cells to HUVECs and inhibits the increase in intercellular adhesion molecule-1 and vascular cell adhesion molecule-1. Moreover, SelS overexpression regulates TNF-*α*-induced inflammatory factors including interleukin-1*β*, interleukin-6, interleukin-8, and monocyte chemotactic protein-1 and attenuates the TNF-*α*-induced activation of p38 mitogen-activated protein kinase (MAPK) and nuclear factor-*κ*B (NF-*κ*B) pathways. Conversely, the knockdown of SelS with siRNA results in an enhancement of TNF-*α*-induced injury in HUVECs. These findings suggest that SelS protects endothelial cells against TNF-*α*-induced dysfunction by inhibiting the activation of p38 MAPK and NF-*κ*B pathways and implicates it as a possible modulator of vascular inflammatory diseases.

## 1. Introduction

The vascular endothelium is regarded as a dynamic organ between blood vessels and circulating blood and plays a crucial role in the cardiovascular system, including the control of fibrinolysis and coagulation, regulation of vascular tone, inflammatory responses and angiogenesis processes, and synthesis and secretion of vasoactive substances [1]. In the healthy state, vascular equilibrium is regulated by a strict balance between agonist and antagonist substances secreted by the endothelium. One of the major vasodilatory substances produced by the endothelium is nitric oxide (NO). NO is a potent relaxing factor that plays a fundamental role in the maintenance of vasomotor function. In addition, it inhibits leukocyte adhesion, platelet aggregation, vascular smooth muscle cells (VSMCs) proliferation, and extracellular matrix secretion [2]. Reduced NO and an imbalance between NO and constriction factors such as endothelin-1 (ET-1) and angiotensin have been implicated in impaired endothelial function [3]. When abnormal vascular homeostasis occurs, injured endothelial cells synthesize and release various kinds of proinflammatory factors and adhesion molecules, such as interleukin-6 (IL-6), interleukin-1*β* (IL-1*β*), interleukin-8 (IL-8), monocyte chemotactic protein-1 (MCP-1), intercellular adhesion molecule-1 (ICAM-1), and vascular cell adhesion molecule-1 (VCAM-1), which facilitate the recruitment, adhesion, and migration of circulating leukocytes to vascular endothelial surfaces and exacerbate inflammatory damage to the endothelium [4].

Several studies have reported a strong relationship between inflammation and the development of endothelial dysfunction [4, 5]. Tumor necrosis factor-*α* (TNF-*α*), one of the prototype proinflammatory cytokines, is highly expressed during a variety of inflammatory conditions and is elevated in the arteries and plasma of humans and animals with vascular complications [6, 7]. Previous studies have also shown that a high level of TNF-*α* could increase endothelium permeability, disrupt endothelium integrity, and induce cytokine secretion, all of which lead to the progression of vascular damage [8].

There is increasing evidence that endothelial dysfunction is crucial for the initiation and progression of primitive atherosclerosis and other forms of cardiovascular diseases including peripheral artery disease, chronic heart failure, hypertension, and coronary artery disease [9]. In this sense, endothelial function is considered as an important predictor of future cardiovascular events for individuals with cardiovascular diseases [10]. Thus, the treatment of endothelial dysfunction is imperative as it promises to reduce cardiovascular risk.

Selenoprotein S (SelS), a member of the selenoprotein family, is located on the endoplasmic reticulum and cell membranes and is expressed in various organs and cells [11]. SelS is involved in the reduction of endoplasmic reticulum stress, resistance to oxidative stress, regulation of inflammation, and glycolipid metabolism [12–15]. SelS has been reported as a receptor for serum amyloid A (SAA), which is an acute inflammatory response protein [16]. Accordingly, the inhibition of SelS is accompanied with increased SAA in lipopolysaccharide- (LPS-) induced HepG2 cells [17]. Fradejas et al. [18] have reported that SelS is markedly increased by the induction of inflammatory stimuli in the brain tissue of C57BL/6 mice, while its inhibition further increases the expression of IL-1*β* and IL-6 in LPS-induced human and mouse astrocytes. These reports indicate that SelS is strongly associated with the regulation of inflammation. However, the molecular mechanisms and effects of SelS on inflammation-induced endothelial damage remain unclear. To address this issue, this study was designed to clarify the biological effects of SelS on TNF-*α*-induced endothelial injury and illustrate the intercellular signaling cascades. We reveal, for the first time, that SelS could regulate TNF-*α*-induced endothelial dysfunction, suggesting that SelS might be a new biomarker for preventing vascular inflammatory disease.

## 2. Materials and Methods

### 2.1. Cell Culture

Human umbilical vein endothelial cells (HUVECs) and THP-1 monocytes were obtained from the American Type Culture Collection. The cells were separately cultured in RPMI-1640 medium containing 10% fetal bovine serum (Hyclone, USA), 100 U/ml penicillin, and 100 *μ*g/ml streptomycin at 37°C and 5% CO_2_. The medium was changed every two days, and the cells were ready for use when they reached 80–90% confluence. The endothelial cells were seeded into 6-well plates and treated with or without TNF-*α* (10 ng/ml, Sigma, USA) at different time points. In some experiments, the cells were pretreated with an inhibitor of the p38 MAPK pathway (SB203580, Selleckchem, USA) or an inhibitor of the NF-*κ*B pathway (S2882, Selleckchem, USA) for 1 h.

### 2.2. Animals

Six-week-old male low-density lipoprotein receptor (LDLR) knockout (KO) mice were purchased from Ireland Matt Technology Co. Ltd. (Suzhou Industrial Park, China) and housed in a specific pathogen-free facility at the Animal Center of Dalian Medical University, China. All animal experiments were approved by the ethical committee of animal experiments in Dalian Medical University (Dalian, China) and followed the Care and Use of Laboratory Animals guidelines sanctioned by the National Institutes of Health. The LDLR-KO mice were randomly divided into control and treatment groups (six mice per group). The control group was given a regular chow (RC) diet, whereas the treatment group was provided with a high-fat diet (HFD, including 10% lard and 2% cholesterol). The mice were fed for 16 weeks during which their food intake, water intake, and body weight were recorded. After the feeding period, sodium pentobarbital solution was administered via an intraperitoneal injection. The LDLR-KO mice were then sacrificed and their thoracic aorta segments were harvested and fixed in neutral formalin. The tissues were then embedded in paraffin and prepared for staining.

### 2.3. Identification of pcDNA3.1-SelS Recombinant Plasmid

A segment of the human SelS gene (GenBank: NM_018445.5) containing 1102 bp (from 104 bp to 1205 bp) was synthesized by Sangon Biotech Co. Ltd. (Shanghai, China). A BamHI cleavage site and a His-tag protein coding sequence were added to the 5′ end, and an EcoRI cleavage site was added to the 3′ end. After EcoRI and BamHI cleavage, the SelS segment was combined with a pcDNA3.1 (+) expression vector by a T4 DNA ligase. The pcDNA3.1-SelS recombinant plasmid was then constructed, and its identification has been previously completed by our study group [19].

### 2.4. Transfection with pcDNA3.1-SelS Recombinant Plasmid or SelS siRNAs

HUVECs were seeded into 6-well plates and prepared for transfection using Lipofectamine 3000 reagent (Invitrogen, USA). Briefly, 2 *μ*g of empty vector plasmid or pcDNA3.1-SelS recombinant plasmid was mixed with 5 *μ*l of Lipofectamine 3000 in serum-free medium. The mixture was dispensed into the wells for incubation. After a 6 h incubation, the cells were cultured in fresh medium for another 24 h and used for the subsequent assays. SelS siRNAs and negative siRNA (the base sequences are provided in Supplementary Table 1) were designed and synthesized by GenePharma Co. Ltd. (Shanghai, China). The steps for the transfection of the SelS siRNAs were the same as for the transfection of the pcDNA3.1-SelS plasmid, and the negative siRNA (Neg.RNA) served as a negative control.

### 2.5. Cell Viability Measurement

The viability of endothelial cells was assessed with a cell counting kit-8 (CCK-8) according to manufacturer's instructions (Dojindo, Kumamoto, Japan). Briefly, the transfected HUVECs were seeded into 96-well plates and treated with TNF-*α*. The CCK-8 solution was added into the cells and incubated at 37°C for 2 h. The absorbance at 490 nm was measured using a microplate reader (NanoDrop, USA). The results were expressed as the percentage of cell viability with respect to control absorbance.

### 2.6. Nitric Oxide Assays

The nitrite concentrations in the medium of different treatment groups which represent NO levels were analyzed using the nitric oxide assay kit according to manufacturer's instructions (KeyGen Biotech, Nanjing, China). The absorbance of the samples was measured using a microplate reader (NanoDrop, USA) at a 550 nm wavelength, and concentration of the samples was determined.

### 2.7. Oxygen-Free Radical Test

HUVECs in different groups were stimulated with TNF-*α* and then incubated with 2′, 7′-dichlorodihydrofluorescein diacetate (2′, 7′-DCFH-DA, Sigma, USA) for 30 min. After washing twice with phosphate-buffered saline (PBS), the levels of cellular reactive oxygen species (ROS) were evaluated using a fluorescence microscope (Leica, Germany). To quantify the results, the photographs were observed under the same exposure condition, and the fluorescence mean densities were estimated using the Image Pro Plus 6.0 software (Microsoft Media Cybernetics, Bethesda, MD, USA).

### 2.8. Cell Adhesion Test

Monocyte adhesion to endothelial cells was determined using fluorescence-labeled THP-1 cells as described in previous studies [20, 21]. In brief, the transfected HUVECs were grown to confluence in 96-well plates and treated with TNF-*α*. The cells were gently washed with serum-free medium, and calcein AM-labeled THP-1 cells (5 × 10^4^/ml DMEM medium) (Sigma, USA) were then added to the endothelial cells. After a 1 h incubation, the endothelial cell monolayer was gently rinsed twice to remove unbound monocytes. The adhesion of THP-1 cells in each group was observed with a fluorescence microscope (Leica, Germany). Under the same exposure condition, the values of fluorescence intensity were measured using the Image Pro Plus 6.0 software (Microsoft Media Cybernetics, Bethesda, MD, USA) and normalized to the control.

### 2.9. Real-Time Quantitative PCR

The total RNA of the samples was extracted using the TRIzol reagent (Takara Bio Inc., Dalian, China). Reverse transcription of the total RNA was then performed using a PrimeScript^TM^ cDNA kit (Takara Bio Inc., Dalian, China) according to manufacturer's directions. Real-time quantitative PCR (RT-qPCR) reactions were incubated initially at 95°C for 30 sec, 95°C for 5 sec, and 60°C for 30 sec of 35 cycles using a SYBR Green PCR Master Mix (Takara Bio Inc., Dalian, China) and were carried out with a PCR System 9700 (Applied Biosystems, USA). Three samples of each group were randomly selected for the RT-qPCR experiment. The samples were analyzed in triplicates, and the relative expression of mRNA was determined using glyceraldehyde-3-phosphatedehydrogenase (GAPDH) as an internal control. The relative gene expression was analyzed using the 2^−ΔΔCt^ method. The specific primers are listed in Supplementary Table 2 and the curves of amplification and dissolution of all RT-qPCR experiments are shown in Supplementary Figure 1.

### 2.10. ELISA

The levels of ICAM-1 and VCAM-1 in the supernatants of cells with different treatments were measured using a commercially available kit (Senbeijia Bio Inc., Nanjing, China) according to manufacturer's instructions. The absorbance of the samples was read on a microplate reader (NanoDrop, USA) at 450 nm.

### 2.11. Western Blot

The lysates of treated cells were reconstituted with loading buffer and run by sodium dodecyl sulfate polyacrylamide gel electrophoresis. Nitrocellulose membranes (Millipore, USA) containing the transferred proteins were soaked in blocking buffer for 2 h and then separately incubated at 4°C overnight with the following primary antibodies: SelS (Sigma, USA), endothelial nitric oxide synthase (eNOS; Proteintech, Wuhan, China), p-c-jun (Proteintech, Wuhan, China), c-jun (Proteintech, Wuhan, China), p-p38 MAPK (Abcam, USA), p38 MAPK (Abcam, USA), inhibitory kappa B *α* kinase *β* (IKK*β*, CST, USA), p-IKK*β* (CST, USA), inhibitory kappa B *α* (I*κ*B*α*, CST, USA), p-I*κ*B*α* (CST, USA), NF-*κ*B p65 (Bioworld, USA), Lamin B (Proteintech, Wuhan, China), and GAPDH (Proteintech, Wuhan, China). After washing for 30 min, the nitrocellulose membranes were then incubated with horseradish peroxidase-conjugated secondary antibody for 1 h at room temperature. The membranes were subsequently treated with enhanced chemiluminescence (ECL, Thermo Scientific, USA) to develop the protein bands, and images were captured using the ChemiDoc MP Imaging System (Bio-Rad, USA). Quantitative analysis of the band intensities was performed with the Quantity One 4.52 software program (Bio-Rad, USA). Lamin B and GAPDH were used as internal controls.

### 2.12. Immunohistochemistry

The sections were dewaxed, hydrated, and microwaved to boiling point in citrate buffer to restore the tissue antigens. Each section was treated with 3% H_2_O_2_ to block endogenous peroxidase activity. After rinsing in PBS, the sections were incubated with bull serum albumin (Beyotime, Shanghai, China) to remove nonspecific antigens. The specimens were then treated with the primary antibody of SelS (1 : 200, Sigma, USA) and incubated at 4°C overnight. Subsequently, the specimens were treated with secondary antibody for 30 min. After treating with freshly constituted diaminobenzidine (ZsBio, Beijing, China), each section was then stained with hematoxylin and rinsed three times. Finally, the sections were dehydrated, rendered transparent, mounted, and dried. The sections were observed and photographed under the same condition using an inverted microscope (Leica, Germany). The mean intensities of the targeted proteins were analyzed semiquantitatively using the Image Pro Plus 6.0 software program (Microsoft Media Cybernetics, Bethesda, MD, USA).

### 2.13. Statistical Analysis

The data were analyzed using SPSS 19.0 (IBM, Armonk, NY, USA) and expressed as mean ± standard deviation (SD). The difference between groups was analyzed with one-way analysis of variance (ANOVA) or the unpaired Student's *t*-test, and *p* values less than 0.05 were considered statistically significant.

## 3. Results

### 3.1. HFD and TNF-*α* Induce Elevated SelS Expression in LDLR-KO Mice and HUVECs, Respectively

**Figure 1:**
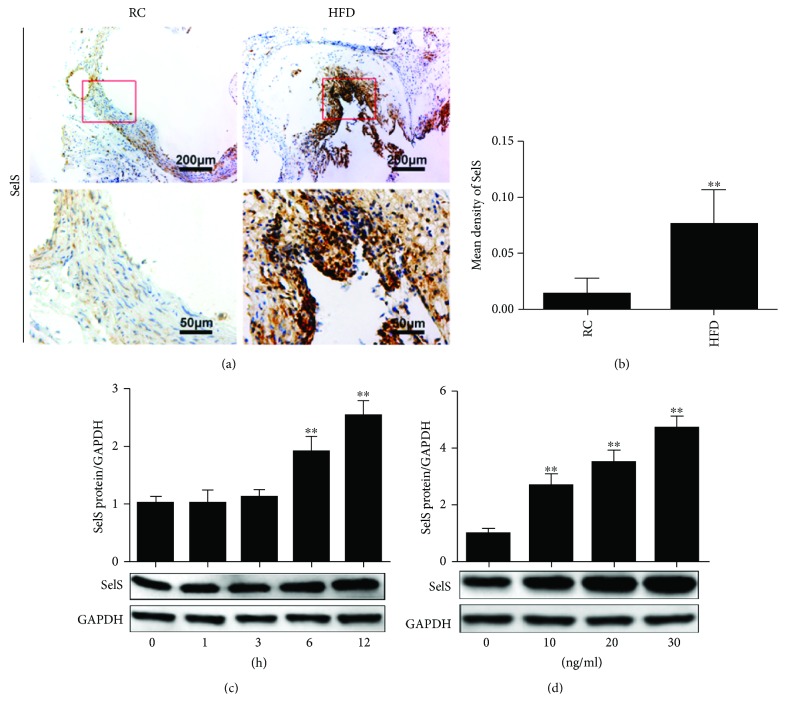
Induction and detection of SelS in LDLR-KO mice and TNF-*α*-treated HUVECs. (a) Representative images of SelS expression detected by immunohistochemistry, and representative images captured at 10x and 40x magnifications. (b) Mean density of SelS analyzed in different groups with the semiquantitative method using the Image Pro Plus 6.0 software. The sections from six mice of each group were observed and three different fields of each slice were randomly selected. (c) Expression of SelS tested in HUVECs at different time points with TNF-*α* (10 ng/ml) stimulation. (d) SelS expression determined in HUVECs with different concentrations of TNF-*α* treatment (for 6 h of incubation). The results are representative of triplicate independent experiments and are presented as mean ± SD, (*n* = 3). ^∗∗^ *p* < 0.01 versus control. LDLR: low-density lipoprotein receptor; KO: knockout; HUVECs: human umbilical vein endothelial cells; RC: regular chow; HFD: high-fat diet.

To investigate the relationship between SelS and endothelial dysfunction, we first explored the levels of SelS in the intima of the thoracic aorta of LDLR-KO mice. As shown in Figures 1(a) and 1(b), the immunohistochemistry staining revealed a significant increase in SelS expression in the aortic intima of LDLR-KO mice fed with HFD. Conversely, LDLR-KO mice fed with RC expressed relatively low levels of SelS (Figures 1(a) and 1(b)). In an *in vitro* study, we investigated the expression levels of SelS after treating HUVECs with TNF-*α*. It was observed that TNF-*α* significantly induced SelS expression in both a time- and dosage-dependent manner (Figures 1(c) and 1(d)). These findings suggest the involvement of SelS in aortic intima damage, and the induction of SelS may be associated with endothelium injury caused by TNF-*α* stimulation.

### 3.2. Transfection of HUVECs with pcDNA3.1-SelS Plasmid or SelS siRNAs and Selection of Transfectant

**Figure 2:**
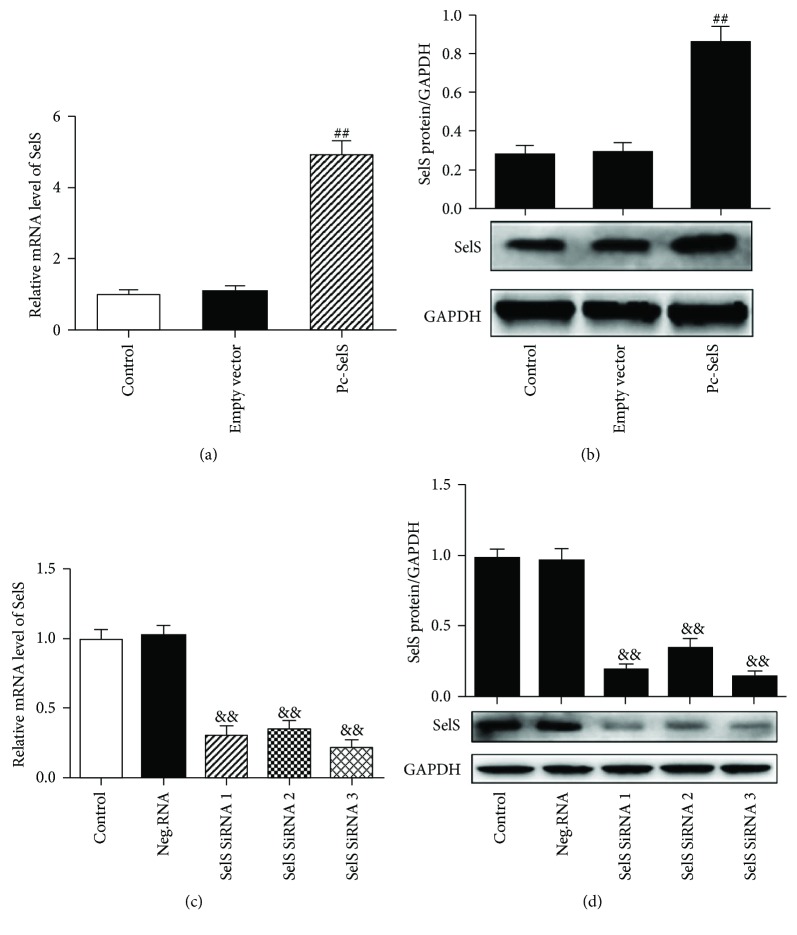
Upregulation and knockdown of SelS in HUVECs. HUVECs with pcDNA3.1-SelS plasmid transfection confirmed by RT-qPCR (a) and by the western blot (b). The mRNA levels (c) and protein expression (d) of SelS determined following the transfection with SelS siRNAs or negative siRNA in HUVECs. The cells were transfected with pcDNA3.1-SelS recombinant plasmid or SelS siRNAs for 30 h. The results are representative of six independent experiments and are presented as mean ± SD (*n* = 6). ^##^ *p* < 0.01 versus empty vector; ^&&^ *p* < 0.01 versus negative siRNA. E-vector: empty vector; Pc-SelS: pcDNA3.1-SelS plasmid; Neg.RNA: negative siRNA.

The transfection technique was employed to explore the functional role of SelS. HUVECs were transfected with either pcDNA3.1-SelS plasmid or SelS siRNAs. The successful overexpression or inhibition of SelS was confirmed by the RT-qPCR and western blot techniques. Both mRNA and protein levels of SelS were, respectively, increased by approximately 4.94-fold and 3.12-fold, respectively, in HUVECs transfected with the pcDNA3.1-SelS plasmid (Figures 2(a) and 2(b)). In contrast, HUVECs transfected with SelS siRNAs demonstrated an inhibition of both mRNA and protein expressions of SelS by SelS siRNA 3, with a reduction of 80% and 88%, respectively (Figures 2(c) and 2(d)). Consequently, SelS siRNA 3 transfection was selected for subsequent experiments.

### 3.3. SelS Enhances Endothelial Cell Viability after TNF-*α* Treatment

**Figure 3:**
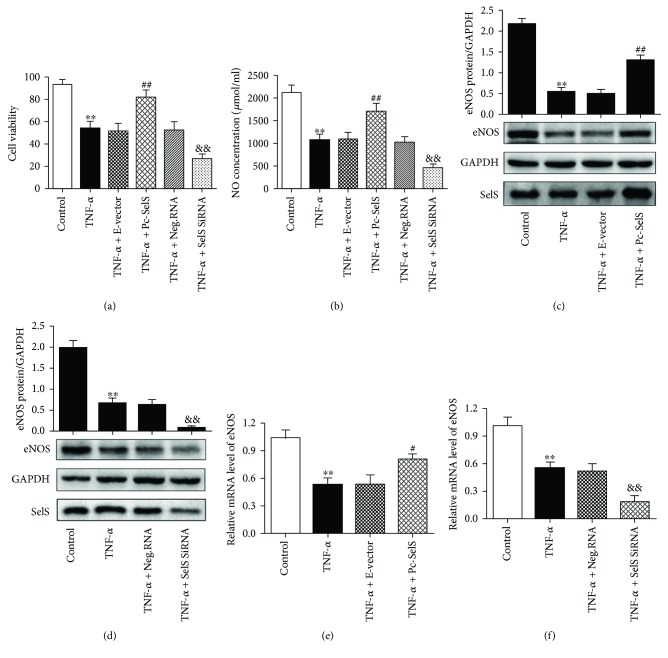
Effect of SelS on the viability of HUVECs and NO and eNOS expressions. (a) Viability of the transfected HUVECs after a 12 h stimulation with TNF-*α* (100 ng/ml), tested using the CCK-8 method. (b) The concentrations of NO in transfected HUVECs with 6 h TNF-*α* (10 ng/ml) treatment, determined using the nitrate reductase assay. The protein expression of eNOS in 10 ng/ml TNF-*α*-induced HUVECs after transfection with SelS plasmid (c) or SelS siRNA (d) examined by the western blot. The mRNA levels of eNOS in 10 ng/ml TNF-*α*-induced HUVECs after transfection with SelS plasmid (e) or SelS siRNA (f). The cells were transfected with pcDNA3.1-SelS recombinant plasmid or SelS siRNAs for 30 h. The eNOS expression was tested after 6 h TNF-*α* treatment. The results are representative of triplicate independent experiments and are presented as mean ± SD, (*n* = 3). ^∗∗^ *p* < 0.01 versus control; ^#^ *p* < 0.05 versus empty vector; ^##^ *p* < 0.01 versus empty vector; ^&&^ *p* < 0.01 versus negative siRNA. CCK-8: cell counting kit-8; NO: nitric oxide; eNOS: endothelial nitric oxide synthase; E-vector: empty vector; Pc-SelS: pcDNA3.1-SelS plasmid; Neg.RNA: negative siRNA.

Increasing evidence has demonstrated that TNF-*α* reduces endothelium viability and promotes its apoptosis, which contributes to the development of cardiovascular diseases. To investigate the role of SelS in endothelial cells viability, we treated the HUVECs with 100 ng/ml TNF-*α* and assessed their viability with CCK-8. The expression of SelS was determined in the experiment and shown in Supplementary Figure 2. The viability of TNF-*α*-treated HUVECs was strongly enhanced in SelS plasmid transfection compared with transfection with an empty vector plasmid (Figure 3(a)). However, SelS siRNA transfection further reduced endothelial cell viability after TNF-*α* treatment (Figure 3(a)). This observation reveals the protective role of SelS in TNF-*α*-associated endothelial damage.

### 3.4. SelS Increases NO and eNOS Levels in TNF-*α*-Treated HUVECs

In order to further observe the effects of SelS on endothelium relaxation, we determined the levels of NO and eNOS in the HUVECs using different treatments. SelS expression was tested in the experiments and shown in Supplementary Figure 2. It was observed that the upregulation of SelS relieved TNF-*α*-induced reduction in NO production (Figure 3(b)). In contrast, the knockdown of SelS in the endothelial cells further reduced TNF-*α*-induced NO level (Figure 3(b)). At the protein level, eNOS was significantly enhanced by SelS overexpression, but the inhibition of SelS further reduced the level of eNOS in the TNF-*α*-treated endothelial cells (Figures 3(c) and 3(d)). Additionally, compared with transfection with the empty vector plasmid, the mRNA expression of eNOS was significantly increased in SelS plasmid transfection (Figure 3(e)). However, SelS siRNA transfection significantly reduced the level of TNF-*α*-induced eNOS in the HUVECs (Figure 3(f)). These results suggest that SelS enhances the levels of NO and eNOS in TNF-*α*-treated endothelial cells.

### 3.5. SelS Reduces Elevated ET-1 and ROS Levels Caused by TNF-*α* Induction

**Figure 4:**
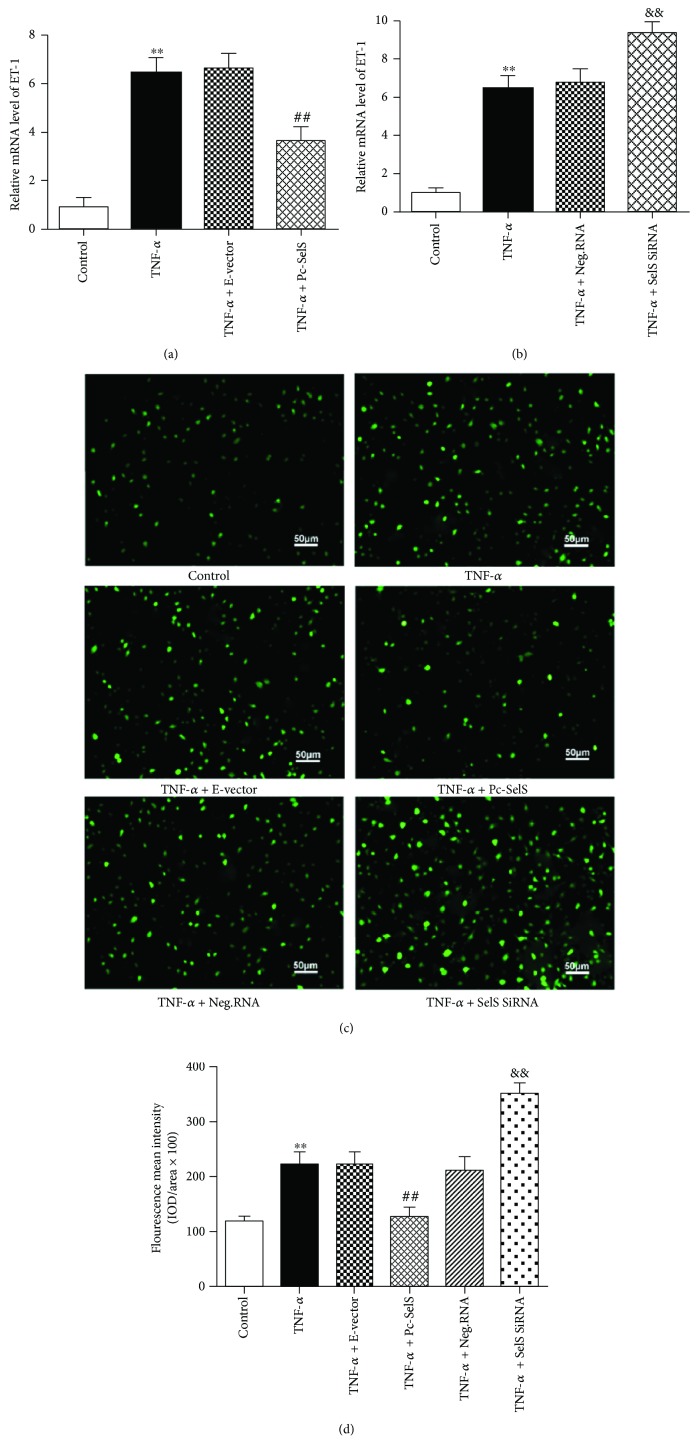
Effect of SelS on the levels of ET-1 and ROS in TNF-*α*-treated HUVECs. The mRNA levels of ET-1 detected in HUVECs transfected with SelS plasmid (a) or SelS siRNA (b) after 10 ng/ml TNF-*α* induction. (c) The levels of cellular ROS measured in different groups with 10 ng/ml TNF-*α* treatment using fluorescent probe method and (d) the corresponding fluorescent mean intensity evaluated by the Image Pro Plus 6.0 software. The cells were transfected with pcDNA3.1-SelS recombinant plasmid or SelS siRNAs for 30 h. The levels of ET-1 and ROS were tested after 6 h TNF-*α* treatment. The results are representative of triplicate independent experiments and are presented as mean ± SD, (*n* = 3). ^∗∗^ *p* < 0.01 versus control; ^##^ *p* < 0.01 versus empty vector; ^&&^ *p* < 0.01 versus negative siRNA. ROS: reactive oxygen species; ET-1: endothelin-1; E-vector: empty vector; Pc-SelS: pcDNA3.1-SelS plasmid; Neg.RNA: negative siRNA.

ET-1 is a potent vasoconstrictor, and regulating its level of expression is crucial to maintaining endothelial function. Similarly, ROS is highly expressed in the endothelium under the stressed condition and facilitates vascular endothelium damage. We, therefore, investigated the effect of SelS on ET-1 and ROS with RT-qPCR and fluorescence staining, respectively. In the transcription state, transfection with SelS plasmid reduced TNF-*α*-induced ET-1 expression in HUVECs, compared with empty vector transfection (Figure 4(a)). HUVECs transfected with SelS siRNA had an increased mRNA expression of ET-1 (Figure 4(b)). Similarly, our fluorescence staining revealed that intracellular ROS levels were largely increased in HUVECs after TNF-*α* treatment (Figures 4(c) and 4(d)). However, the upregulation of SelS inhibited the increase in TNF-*α*-induced ROS production. In contrast, the knockdown of SelS in the endothelial cells further elevated the production of ROS induced by TNF-*α* (Figures 4(c) and 4(d)). Collectively, these outcomes indicate that SelS protects the endothelium by regulating ET-1 and ROS expressions in TNF-*α*-induced endothelial damage.

### 3.6. SelS Suppresses TNF-*α*-Induced Adhesion Molecules and the Adhesion of THP-1 Cells to Endothelial Cells

**Figure 5:**
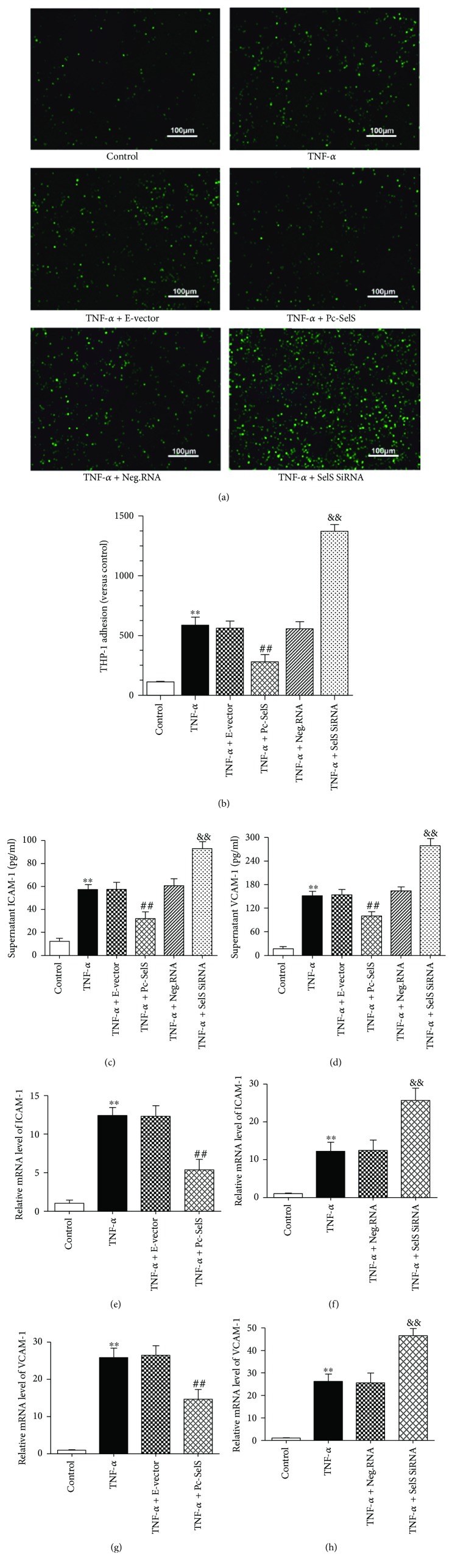
Effect of SelS on THP-1 cells adhesion and adhesion factors. (a) Adhesion of THP-1 cells to HUVECs shown by the fluorescent probe method and (b) the corresponding fluorescence intensity analyzed using Image Pro Plus 6.0 software. The production of ICAM-1 (c) and VCAM-1 (d) detected in the supernatant of cultures with 10 ng/ml TNF-*α* treatment by ELISA. The mRNA levels of ICAM-1 estimated in 10 ng/ml TNF-*α*-induced cells after transfection with SelS plasmid (e) or SelS siRNA (f). The mRNA levels of VCAM-1 determined in SelS plasmid (g) or SelS siRNA (h) transfected cells after 10 ng/ml TNF-*α* induction (for 6 h). The cells were transfected with pcDNA3.1-SelS recombinant plasmid or SelS siRNAs for 30 h. The results are representative of triplicate independent experiments and are presented as mean ± SD, (*n* = 3). ^∗∗^ *p* < 0.01 versus control; ^##^ *p* < 0.01 versus empty vector; ^&&^ *p* < 0.01 versus negative siRNA. ICAM-1: intercellular adhesion molecule-1; VCAM-1: vascular cell adhesion molecule-1; E-vector: empty vector; Pc-SelS: pcDNA3.1-SelS plasmid; Neg.RNA: negative siRNA.

In trauma, the trafficking of leukocytes to the endothelium is among the initial stages in endothelial injury. Therefore, we examined the effect of SelS on the TNF-*α*-induced adhesion of THP-1 cells to endothelial cells. We observed via fluorescence staining that SelS plasmid transfection significantly suppressed the TNF-*α*-induced adhesion of THP-1 cells (Figures 5(a) and 5(b)). However, the reverse was noticed in SelS siRNA transfection, where a significant increase in the adhesion of THP-1 cells to HUVECs was noted (Figures 5(a) and 5(b)). Because several molecules mediate leukocyte adhesion to endothelial cells, we examined the effects of SelS on ICAM-1 and VCAM-1 expressions with ELISA and RT-qPCR, respectively. TNF-*α* generally augmented the levels of ICAM-1 and VCAM-1 compared with the control group (Figures 5(c)–5(h)). However, SelS overexpression significantly reduced TNF-*α*-induced increase in the production of ICAM-1 and VCAM-1, while the inhibition of SelS notably enhanced the TNF-*α*-induced expression of ICAM-1 and VCAM-1 (Figures 5(c)–5(h)). These results indicate that SelS has the potential to reduce leukocyte adhesion by inhibiting adhesion molecules.

### 3.7. SelS Reduces the Expression of Chemokines and Cytokines Induced by TNF-*α*

**Figure 6:**
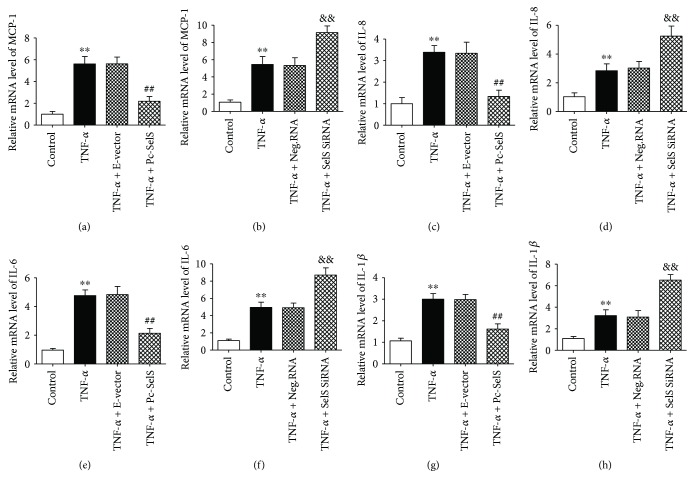
Influence of SelS on inflammatory factors. The effect of SelS on the mRNA level of MCP-1 (a), IL-8 (c), IL-6 (e), and IL-1*β* (g) was determined in TNF-*α*-treated cells (10 ng/ml) by means of SelS transfection. The mRNA levels of MCP-1 (b), IL-8 (d), IL-6 (f), and IL-1*β* (h) in transfected cells with 10 ng/ml TNF-*α* treatment (for 6 h), estimated via RT-qPCR. The cells were transfected with pcDNA3.1-SelS recombinant plasmid or SelS siRNAs for 30 h. The results are representative of triplicate independent experiments and are presented as mean ± SD, (*n* = 3). ^∗∗^ *p* < 0.01 versus control; ^##^ *p* < 0.01 versus empty vector; ^&&^ *p* < 0.01 versus negative siRNA. MCP-1: monocyte chemoattractant protein-1; IL-8: interleukin-8; IL-6: interleukin-6; IL-1*β*: interleukin-1*β*; E-vector: empty vector; Pc-SelS: pcDNA3.1-SelS plasmid; Neg.RNA: negative siRNA.

Chemokines, such as MCP-1 and IL-8, are responsible for recruiting leukocytes and for their migration to the subintimal layer of injured endothelium. The influence of SelS on TNF-*α* was, therefore, investigated by measuring the expression levels of MCP-1 and IL-8 using RT-qPCR. At the transcription stage, mRNA levels of MCP-1 and IL-8 were significantly enhanced by TNF-*α* stimulation but were reduced in SelS overexpression (Figures 6(a) and 6(c)). Compared with negative siRNA transfection, the levels of MCP-1 and IL-8 were elevated in SelS knockdown cells (Figures 6(b) and 6(d)). In addition, we investigated the effects of SelS on the proinflammatory cytokines, IL-6 and IL-1*β*, which exacerbate endothelial damage, and they were elevated by TNF-*α* (Figures 6(e)–6(h)). Similar to the observation made in MCP-1 and IL-8 production, the upregulation of SelS reduced the mRNA expression of IL-6 and IL-1*β* in TNF-*α*-treated HUVECs. However, the expression of these cytokines was enhanced in SelS knockdown cells (Figures 6(e)–6(h)). The inhibition of these molecules by SelS suggests its antagonizing effect against proinflammatory chemokines and cytokines.

### 3.8. SelS Inhibits the Activation of the TNF-*α*-Induced p38 MAPK Pathway

**Figure 7:**
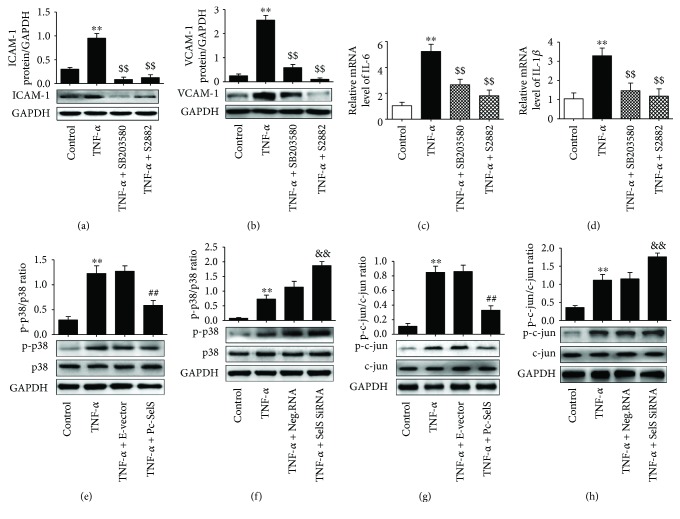
Effect of SelS on the p38 MAPK pathway. The expression of ICAM-1 (a) and VCAM-1 (b) in cells treated with SB203580 (an inhibitor of the p38/MAPK pathway) or S2882 (an inhibitor of the NF-*κ*B pathway), prior to 10 ng/ml TNF-*α* treatment, detected by the western blot. The mRNA levels of IL-6 (c) and IL-1*β* (d) in HUVECs treated with SB203580 or S2882 before 10 ng/ml TNF-*α* induction. (e) and (f) The levels of phosphorylated p38 MAPK and p38 MAPK tested in the transfected cells after 10 ng/ml TNF-*α* stimulation. The expression of phosphorylated c-jun and c-jun in SelS plasmid (g) or SelS siRNA (h) transfection following 10 ng/ml TNF-*α* treatment and determined by the western blot. The cells were transfected with pcDNA3.1-SelS recombinant plasmid or SelS siRNAs for 30 h. The dosage used for inhibitors SB203580 and S2882 was 20 *μ*M and 10 *μ*M, respectively. The results are representative of triplicate independent experiments and are presented as mean ± SD, (*n* = 3). ^∗∗^ *p* < 0.01 versus control; ^##^ *p* < 0.01 versus empty vector; ^&&^ *p* < 0.01 versus negative siRNA; ^$$^ *p* < 0.01 versus TNF-*α* treatment. E-vector: empty vector; Pc-SelS: pcDNA3.1-SelS plasmid; Neg.RNA: negative siRNA.

As shown in Figures 7(a)–7(d), when HUVECs were treated with SB203580 (a p38 MAPK pathway inhibitor) or S2882 (a NF-*κ*B pathway inhibitor), the elevation of ICAM-1, VCAM-1, IL-6, and IL-1*β* by TNF-*α* induction was significantly inhibited, suggesting that the production of these adhesion molecules and proinflammatory factors partly depends on the activation of the NF-*κ*B and p38 MAPK signaling pathways. Consequently, we first evaluated the role of SelS in the activation of p38 MAPK signaling pathways. The levels of phosphorylated p38 MAPK were upregulated after treatment with TNF-*α* for 30 min (Figures 7(e) and 7(f)). Subsequently, there was an increase in its downstream phosphorylated c-jun (a subunit of activator protein-1) in endothelial cells treated with TNF-*α* for 1 h (Figures 7(g) and 7(h)). Conversely, the upregulation of SelS significantly reduced the TNF-*α*-induced activation of phosphorylated p38 MAPK and phosphorylated c-jun, while SelS knockdown further increased TNF-*α*-induced phosphorylated proteins in HUVECs (Figures 7(e)–7(h)).

### 3.9. SelS Inhibits the Activation of the TNF-*α*-Induced NF-*κ*B Pathway

**Figure 8:**
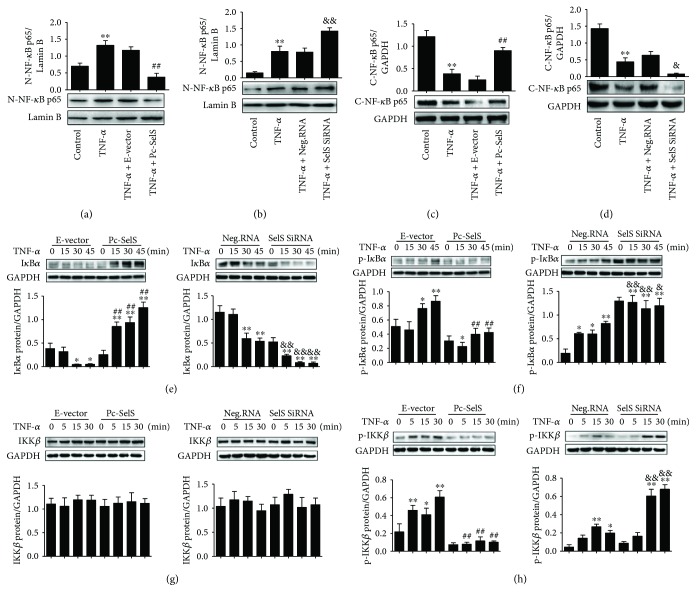
Effect of SelS on the NF-*κ*B pathway. Levels of nuclear NF-*κ*B p65 in SelS plasmid (a) or SelS siRNA (b) transfected HUVECs after 10 ng/ml TNF-*α* treatment, determined by the western blot. The expression of cytoplasmic NF-*κ*B p65 in HUVECs transfected with SelS plasmid (c) or SelS siRNA (d) and treated with 10 ng/ml TNF-*α*. Expression of I*κ*B*α* (e) and phosphorylated I*κ*B*α* (f) detected in different groups at indicated time points after 10 ng/ml TNF-*α* induction. Levels of IKK*β* (g) and phosphorylated IKK*β* (h) detected in HUVECs with different treatments after 10 ng/ml TNF-*α* induction. The cells were transfected with pcDNA3.1-SelS recombinant plasmid or SelS siRNAs for 30 h. The results are representative of triplicate independent experiments and are presented as mean ± SD, (*n* = 3). ^∗^ *p* < 0.05 and ^∗∗^ *p* < 0.01 versus control; ^##^ *p* < 0.01 versus empty vector at corresponding time point; ^&^ *p* < 0.05 and ^&&^ *p* < 0.01 versus negative siRNA at corresponding time points. I*κ*B*α*: inhibitory kappa B *α*; IKK*β*: inhibitor of nuclear factor kappa-B kinase *β*; N-NF-*κ*B p65: nuclear NF-*κ*B p65; C-NF-*κ*B p65: cytoplasmic NF-*κ*B p65; E-vector: empty vector; Pc-SelS: pcDNA3.1-SelS plasmid; Neg.RNA: negative siRNA.

The translocation of NF-*κ*B from the cytoplasm to the nucleus is an essential step for the activation of inflammatory factors. HUVECs pretreated with SelS plasmid or SelS siRNA were incubated with TNF-*α* within a range of indicated time points. Cytoplasmic and nuclear lysates were western blotted for NF-*κ*B p65 to determine the translocation of NF-*κ*B. TNF-*α* treatment increased nuclear NF-*κ*B p65 levels and reduced cytoplasmic NF-*κ*B p65 levels compared with the control group (Figures 8(a)–8(d)). However, SelS overexpression reduced the levels of nuclear NF-*κ*B p65 and enhanced the expression of cytoplasmic NF-*κ*B p65, but the inhibition of SelS further enhanced the expression of TNF-*α*-induced nuclear NF-*κ*B p65 and decreased the levels of cytoplasmic NF-*κ*B p65 (Figures 8(a)–8(d)), indicating that SelS inhibits the translocation of NF-*κ*B from the cytoplasm to the nucleus in HUVECs.

To further explore the mechanisms by which SelS inhibits NF-*κ*B activation, we studied its upstream pathways by investigating the effects of SelS on IKK*β*/I*κ*B*α*. We observed that TNF-*α* treatment rapidly led to the phosphorylation of I*κ*B*α*, degradation of I*κ*B*α*, and phosphorylation of IKK*β* in HUVECs in a time-dependent manner (Figures 8(e)–8(h)). Moreover, the upregulation of SelS markedly inhibited the TNF-*α*-induced phosphorylation of I*κ*B*α* and suppressed the degradation of I*κ*B*α* induced by TNF-*α* (Figures 8(e) and 8(f)). There was no significant difference in the expression of IKK*β* in SelS plasmid transfection or SelS siRNA transfection (Figure 8(g)). However, the phosphorylation of IKK*β* was significantly reduced in cells transfected with the SelS plasmid and was further increased in cells transfected with SelS siRNA (Figure 8(h)), suggesting SelS as a negative regulator of IKK*β*/I*κ*B*α* signaling via the inhibition of I*κ*B*α* and IKK*β* phosphorylation and via the degradation of I*κ*B*α* in endothelial inflammation.

## 4. Discussion

As a biological barrier between the vessel wall and circulating blood, the vascular endothelium is especially crucial for the maintenance of vascular homeostasis [1]. Many risk factors such as smoking, hyperglycemia, and hyperlipidemia may promote the pathogenesis of endothelial dysfunction through the modulation of inflammatory pathways [9]. SelS has been reported to be strongly associated with inflammation [14]. In our *in vivo* study, we observed a significant increase in SelS expression in the aortic intima of LDLR-KO mice fed with HFD, indicating that SelS is closely associated with the formation of arteriosclerosis (AS). However, endothelial dysfunction has been established as a foundation in the initiation and progression of AS, and SelS is highly expressed during endothelial injury. The upregulation of SelS could, therefore, be viewed as a defensive response against AS formation and seems to play a mediatory role in endothelial dysfunction. HUVECs are usually employed to explore the biology of vascular endothelial cells, inflammation, and the onset mechanisms of multiple diseases. We found that TNF-*α* increased SelS expression in HUVECs in a time- and dose-dependent manner (Figure 1), indicating that TNF-*α* regulates SelS expression, and suggesting that SelS plays a critical role in endothelial inflammation.

In order to better understand the molecular mechanisms underlying inflammatory endothelial dysfunction, we examined the effects of SelS on TNF-*α*-induced HUVECs dysfunction. The hallmark of endothelial dysfunction is impaired NO bioavailability. NO is synthesized by eNOS during the conversion of L-arginine to L-citrulline within endothelial cells, and eNOS, a dimeric enzyme, is mainly expressed by vascular endothelial cells [22]. We found that SelS overexpression improved TNF-*α*-induced NO reduction by increasing the level of eNOS (Figure 3). Under normal conditions, inactive eNOS binds to caveolin-1 (Cav-1) and remains in cytoplasmic caveolae. When the concentration of cytoplasmic Ca^2+^ increases, caldesmon (CAM) replaces Cav-1 and binds to eNOS to activate it [23]. However, the upregulation of Cav-1 led to an interruption in the activation of eNOS in the liver of mice [24]. Our previous study found that transfection with SelS plasmid inhibited H_2_O_2_-induced Cav-1 expression in HUVECs [25]. As such, SelS may influence the levels of eNOS by regulating Cav-1 in HUVECs; however, this hypothesis requires more research for verification.

In the vascular system, excessive ROS facilitates the production of superoxide anions (O_2_^•−^) in endothelial cells. O_2_^•−^ easily binds to NO and forms peroxynitrite, compromising vasorelaxation [26]. However, we observed, in this study, that the upregulation of SelS significantly reduced TNF-*α*-induced production of ROS in the endothelial cells (as shown in Figure 4). Our findings suggest that the elevated expression of SelS increases NO levels not only by increasing the levels of eNOS but also by reducing ROS production and indicate that the free radical scavenging effect of SelS contributes to its anti-inflammatory effects. ET-1 is a potent vasoconstrictor peptide that exhibits both prooxidant and proinflammatory properties and accelerates the development of endothelial dysfunction [27]. TNF-*α* induces the excessive production of ET-1 in the endothelium, and excessive ET-1 reduces eNOS expression by influencing the distribution of eNOS between the membrane and mitochondria [28]. Additionally, the characteristic manifestation of endothelial injury is a dysfunctional vasorelaxation caused by a severe imbalance between NO and ET-1. We demonstrated in this study that the elevation of TNF-*α*-induced ET-1 expression in HUVECs is suppressed by SelS overexpression (Figure 4). Optimizing and controlling SelS expression could be explored in endothelial dysfunction to enhance the level of eNOS via inhibition of ET-1 expression and also to modulate ET-1 and NO communication.

Endothelial dysfunction is also characterized by leukocyte accumulation and increased adhesions. When endothelial cells undergo inflammatory activation, an increase in MCP-1 and IL-8 promotes the recruitment of leukocytes, with a corresponding increase in ICAM-1 and VCAM-1, which promotes the adherence of leukocyte to the endothelium and aggravates endothelial damage. Our model confirmed that TNF-*α* mediates the production of ICAM-1, VCAM-1, MCP-1, and IL-8 and enhances leukocyte adhesion to the endothelium (Figures 5 and 6). Notably, these effects were downregulated after SelS overexpression, demonstrating that SelS overexpression reduces leukocyte adhesion by possibly inhibiting its chemotaxis and adhesive molecules during inflammation. It has been established that the elevation of inflammatory cytokines such as IL-1*β* and IL-6 has adverse consequences and could worsen endothelial damage and that limiting their expression implies the reduction of the associated cellular insult. Our study shows that the upregulation of SelS expression attenuates the deleterious effects of IL-1*β* and IL-6 during endothelial inflammation. By extension, SelS appears to regulate the expression of several proinflammatory genes associated with endothelial dysfunction.

We deduced from our study that the expression of targeted cytokines and adhesion factors following endothelial injury induced by TNF-*α* is mediated by the activation of the p38 MAPK pathway. Activator protein-1 (AP-1), a transcription factor, regulates a variety of cytokine expressions and consists of the jun and fos proteins [29]. In most cells, the AP-1 dimer consists of c-jun and c-fos [29]. When TNF-*α* binds to its receptors on the endothelium, p38 MAPK is activated which leads to the phosphorylation of c-jun and c-fos and the subsequent activation of AP-1 [30]. In this study, HUVECs transfected with SelS plasmid inhibited TNF-*α*-induced phosphorylated p38 MAPK and phosphorylated c-jun activation (Figure 7). This suggests that the elevated expression of SelS attenuates TNF-*α*-induced inflammation in endothelial cells by inhibiting the activation of p38 MAPK pathways.

Similarly, treating HUVECs with the NF-*κ*B inhibitor S2882 downregulated ICAM-1, VCAM-1, IL-6, and IL-1*β* in TNF-*α*-treated cells, revealing that TNF-*α* induces dysfunction through activating the NF-*κ*B pathway. In NF-*κ*B signaling, the p50-p65 heterodimer contributes to the regulation of the transcription of inflammatory-related factors [31]. When endothelial cells are inactive, NF-*κ*B remains in the cell cytoplasm and the p65 subunit binds to the protein I*κ*B*α*, covering the p50 subunit, which serves as the nuclear localization signal. However, when endothelial cells are stimulated by TNF-*α*, the phosphorylated inhibitor of IKK*β* causes the phosphorylation of I*κ*B*α* and detachment from NF-*κ*B. NF-*κ*B then migrates from the cytoplasm to the nucleus to regulate gene transcription [31]. This study demonstrated that the upregulation of SelS inhibited TNF-*α*-induced p65 migration from the cytoplasm to the nucleus (Figure 8). In addition, SelS reduced the levels of phosphorylated IkB*α* and phosphorylated IKK*β* in HUVECs in a time-dependent manner, following TNF-*α* treatment, suggesting that SelS suppressed NF-*κ*B translocation by regulating IKK*β*/IkB*α* signaling pathways. Other study revealed that there are two loci bound by NF-*κ*B in the SelS promoter region in HepG2 cells [14], which suggest that NF-*κ*B regulates SelS genes transcription. However, in our study, SelS overexpression regulated NF-*κ*B translocation. By inference, we hypothesize that a feedback loop exists between SelS and NF-*κ*B, in which NF-*κ*B increases SelS transcription, and that conversely elevated SelS expression inhibits the excessive activation of NF-*κ*B.

This is the first study to demonstrate that SelS could protect HUVECs from inflammatory damage by inhibiting the p38 MAPK and NF-*κ*B pathways. Our previous studies have shown that SelS overexpression protects the endothelium from oxidative stress injury by the inhibition of H_2_O_2_-induced Cav-1 [25]. Another study has indicated that SelS overexpression increases the resistance of VSMCs to oxidative stress damage [13]. In addition, SelS may be associated with the pathogenesis and development of diabetic macroangiopathy [19]. In the present study, we have demonstrated that SelS attenuates endothelial dysfunction via the inhibition of inflammatory responses. These results elucidate the mechanism of SelS function in vascular protection. SelS may be considered as a clinical predictive biomarker or a therapeutic option for vascular diseases associated with endothelial dysfunction.

## 5. Conclusion

In summary, we have shown that the upregulation of SelS improves the viability of endothelial cells, enhances the levels of NO and eNOS, reduces the production of ET-1 and ROS, and inhibits the adhesion of TNF-*α*-induced THP-1 cells to endothelial cells. SelS overexpression also reduces the expression of ICAM-1, VCAM-1, MCP-1, IL-8, IL-6, and IL-1*β* mediated by TNF-*α*. In addition, the upregulation of SelS inactivates TNF-*α*-induced NF-*κ*B and p38 MAPK pathways, while the knockdown of SelS leads to an enhancement of inflammatory damage to endothelial cells. Moreover, a feedback signaling may exist between SelS and NF-*κ*B, and the overexpression of SelS attenuates TNF-*α*-induced endothelial dysfunction by inhibiting the p38 MAPK and NF-*κ*B pathways in HUVECs.

## Abbreviations

AS: Atherosclerosis
AP-1: Activator protein-1
CCK-8: Cell count kit-8
Cav-1: Caveolin-1
CAM: Caldesmon
ET-1: Endothelin-1
eNOS: Endothelial nitric oxide synthase
IL-1*β*: Interleukin-1*β*
IL-6: Interleukin-6
IL-8: Interleukin-8
ICAM-1: Intercellular adhesion molecule-1
I*κ*B*α*: Inhibitory kappa B *α*
IKK*β*: Inhibitory kappa B kinase *β*
NO: Nitric oxide
NF-*κ*B: Nuclear factor-*κ*B
p38 MAPK: p38 mitogen-activated protein kinase
ROS: Reactive oxygen species
SelS: Selenoprotein S
SNP: Single nucleotide polymorphism
siRNA: Small interfering RNA
TNF-*α*: Tumour necrosis factor-alpha
VSMCs: Vascular smooth muscle cells
VCAM-1: Vascular cell adhesion molecule-1.

## Supplementary Materials

Supplementary Materials Table S1: the sequences of SelS siRNAs and negative siRNA are shown. The sequences of SelS siRNA 1, SelS siRNA 2, SelS siRNA 3, and negative siRNA are listed in Table S1. The SelS siRNAs were used for SelS knockdown and the negative siRNA was used as a control in liposome transfection experiments. Table S2: the specific primers for RT-qPCR are shown. The sequences of specific primers such as SelS, GAPDH, eNOS, ET-1, ICAM-1, VCAM-1, IL-6, IL-1*β*, MCP-1, and IL-8 are listed in Table S2. The primers were used for RT-qPCR experiments to test gene mRNA levels. The reactions were incubated initially at 95°C for 30 sec, 95°C for 5 sec, and 60°C for 30 sec of 35 cycles and were carried out with a PCR System 9700 (Applied Biosystems, USA). The relative expression of mRNA was determined using GAPDH as an internal control. Figure S1: the dissolve and amplification curves are shown in RT-qPCR experiments. The dissolve and amplification curves of RT-qPCR experiments are listed in Figure S1. The left part of the figure is shown as amplification curves and the right part of the figure is shown as dissolve curves in the RT-qPCR experiments. The curves could reflect the accuracy, credibility, and authenticity of the results. Figure S2: the transfection with pcDNA3.1-SelS recombinant plasmid or SelS siRNA is identified in Figure 3 listed experiments. (a) pcDNA3.1-SelS plasmid transfection and (b) SelS siRNA transfection using in cell viability experiment were determined by western blot. (c) The transfection with pc-SelS plasmid and (d) transfection with SelS siRNA using in NO measurement were tested by western blot. (e) The pc-SelS plasmid transfection or (f) SelS siRNA transfection in eNOS mRNA testing was determined by RT-qPCR.
