# Supplementary material for: Selenoprotein S Attenuates Tumor Necrosis Factor-α-Induced Dysfunction in Endothelial Cells
Source: Mediators Inflamm. 2018 Apr 1;2018:1625414. doi: 10.1155/2018/1625414 (PMC5901950; doi:10.1155/2018/1625414)
Supplement: Supplementary Materials — Table S1: the sequences of SelS siRNAs and negative siRNA are shown. The sequences of SelS siRNA 1, SelS siRNA 2, SelS siRNA 3, and negative siRNA are listed in Table S1. The SelS siRNAs were used for SelS knockdown and the negative siRNA was used as a control in liposome transfection experiments. Table S2: the specific primers for RT-qPCR are shown. The sequences of specific primers such as SelS, GAPDH, eNOS, ET-1, ICAM-1, VCAM-1, IL-6, IL-1β, MCP-1, and IL-8 are listed in Table S2. The primers were used for RT-qPCR experiments to test gene mRNA levels. The reactions were incubated initially at 95°C for 30 sec, 95°C for 5 sec, and 60°C for 30 sec of 35 cycles and were carried out with a PCR System 9700 (Applied Biosystems, USA). The relative expression of mRNA was determined using GAPDH as an internal control. Figure S1: the dissolve and amplification curves are shown in RT-qPCR experiments. The dissolve and amplification curves of RT-qPCR experiments are listed in Figure S1. The left part of the figure is shown as amplification curves and the right part of the figure is shown as dissolve curves in the RT-qPCR experiments. The curves could reflect the accuracy, credibility, and authenticity of the results. Figure S2: the transfection with pcDNA3.1-SelS recombinant plasmid or SelS siRNA is identified in Figure 3 listed experiments. (a) pcDNA3.1-SelS plasmid transfection and (b) SelS siRNA transfection using in cell viability experiment were determined by western blot. (c) The transfection with pc-SelS plasmid and (d) transfection with SelS siRNA using in NO measurement were tested by western blot. (e) The pc-SelS plasmid transfection or (f) SelS siRNA transfection in eNOS mRNA testing was determined by RT-qPCR. [file 1625414.f1.pdf]

Selenoprotein S Attenuates Tumor Necrosis Factor-α Induced Dysfunction in Endothelial Cells

Siyuan Cui[1], Lili Men[1], Yu Li[1], Yingshuo Zhong[1], Shanshan Yu[1], Fang Li[2], Jianling Du[1]

[1] Department of Endocrinology, The First Affiliated Hospital of Dalian Medical University, Dalian 116000, Liaoning, China

[2] Department of Immunology, Dalian Medical University, Dalian 116000, Liaoning, China

The Corresponding authors' email: Fang Li, lifang16@hotmail.com. Jianling Du, dujianlingcn@163.com. Jianling Du is the first corresponding author.

Disclosure statement: The authors have nothing to disclose.

Table S1: the sequences of SelS siRNAs and Negative siRNA were shown.

Designation	Sequence
SelS siRNA 1	sense strand 5'-CCACCUAUGGCUGGUACAUTT-3'
	antisense strand 5'-AUGUACCAGCCAUAGGUGGTT-3'
SelS siRNA 2	sense strand 5'-CCUUCUCUACGUGGUCUUUTT-3'
	antisense strand 5'-AAAGACCACGUAGAGAAGGTT-3'
SelS siRNA 3	sense strand 5'-GGAACCUGAUGUUGUUGUUTT-3'
	antisense strand 5'-AACAACAACAUCAGGUUCCTT-3'
Negative siRNA	sense strand 5'-UUCUCCGAACGUGUCACGUTT-3'
	antisense strand 5'-ACGUGACACGUUCGGAGAATT-3'

Table S2: the specific primers for RT-qPCR were shown.

Gene	Sequences
Human SelS	5'-GTTGCGTTGAATGATGTCTTCCT-3'
	5'-AGAAACAAACCCCATCAACTGT-3'
Human GAPDH	5'-TGACCACAGTCCATGCCATCAC-3'
	5'-CGCCTGCTTCACCACCTTCTT-3'
Human eNOS	5'-CTGAAGGCTGGCATCTGGAA-3'
	5'-CATGTTACTGTGCGTCCACTCTG-3'
Human ET-1	5'-TCCTCTGCTGGTTCCTGACT-3'
	5'-CAGAAACTCACCCCTGTGT-3'
Human ICAM-1	5'-CAAGGTGACCGTGAATGTGC-3'
	5'-CGTGGCTTGTGTGTTCGGTT-3'
Human VCAM-1	5'-CCACATCTACGCTGACAATG-3'
	5'-GAACACTTGACTGTGATCGG-3'
Human IL-6	5'-TACATCCTCGACGGCATCTCA-3'
	5'-GCCATCTTTGGAAGGTTCAGG-3'
Human IL-1β	5'-CCTGTCCTGCGTGTTGAAAG-3'
	5'-GAGAGGTGCTGATGTACCAG-3'
Human MCP-1	5'-GAAGAATCACCAGCAGCAAG-3'
	5'-GAATCCTGAACCCACTTCTG-3'
Human IL-8	5'-AGTTTTGCCAAGGAGTGCTA-3'
	5'- AAAACTTCTCCACAACCCTC-3'

Figure S1: the dissolve and amplification curves were shown in RT-qPCR experiments.

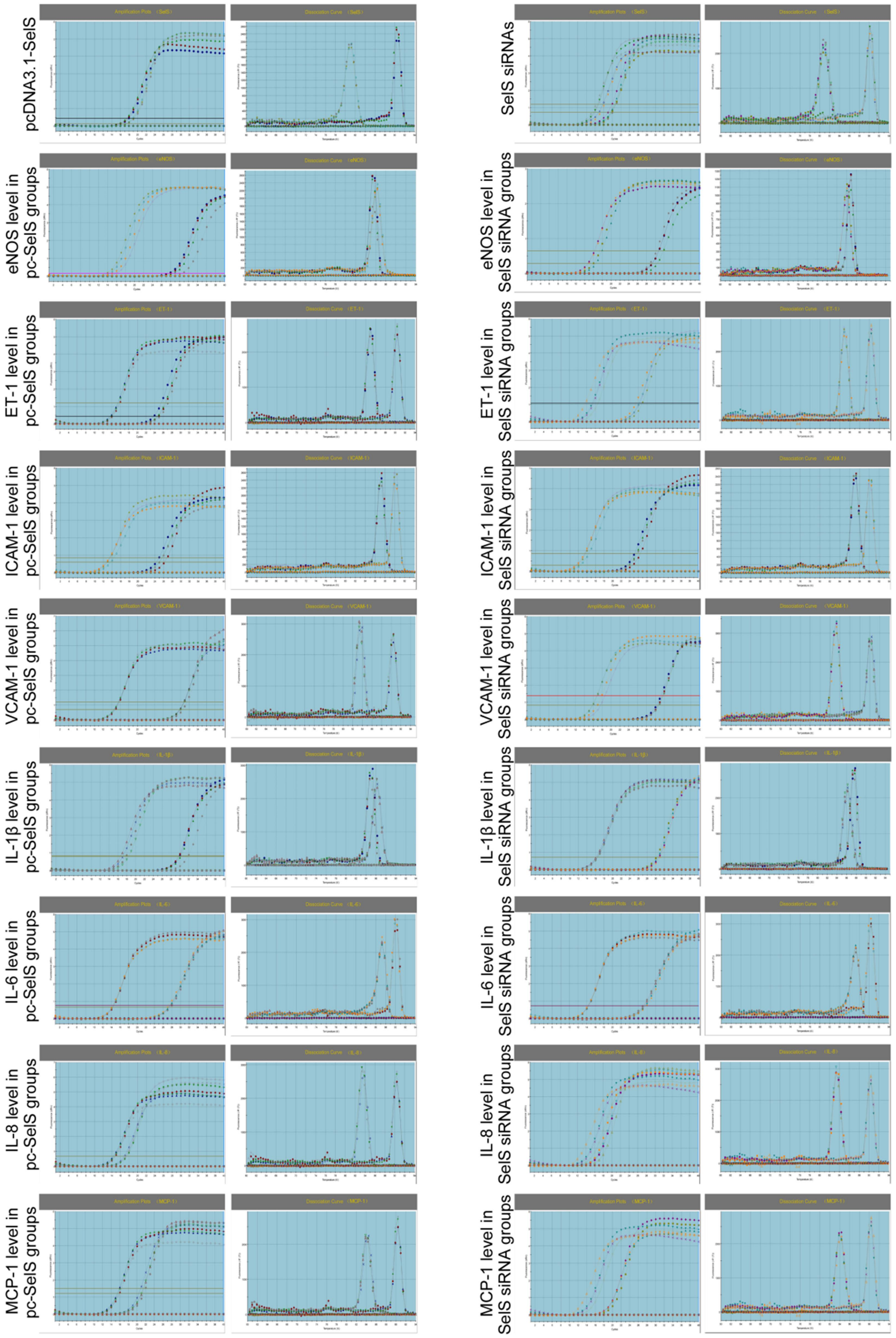

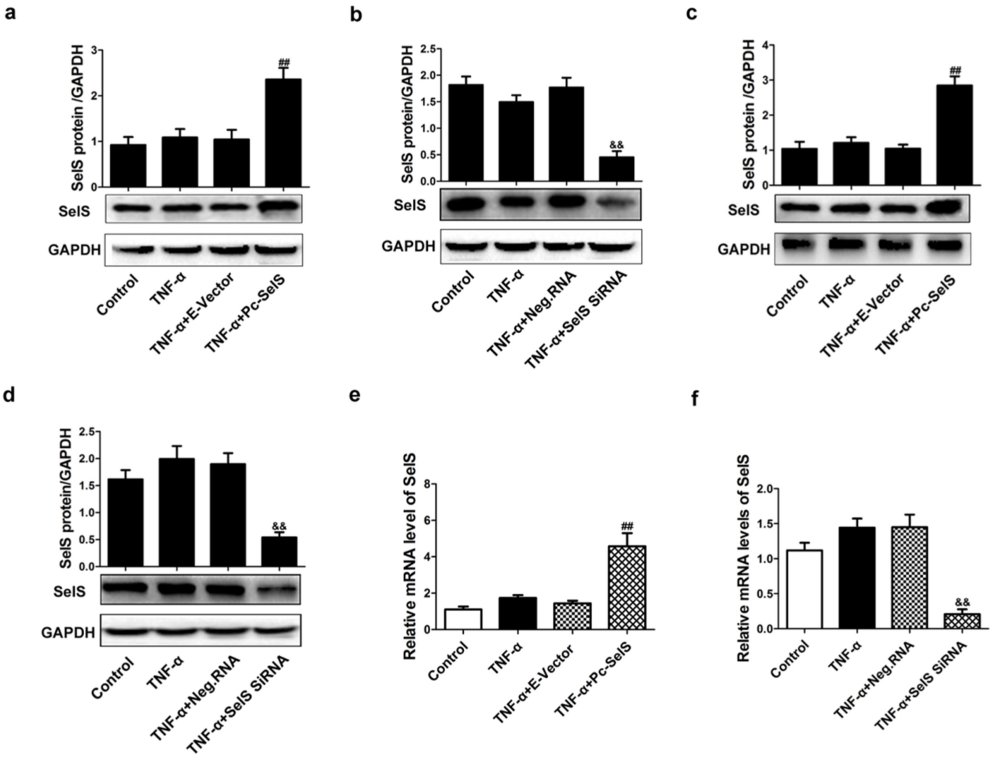

Figure S2: The transfection with pcDNA3.1-SelS recombinant plasmid or SelS siRNA was identified in Figure 3 listed experiments. (a) pcDNA3.1-SelS plasmid transfection and (b) SelS siRNA transfection using in cell viability experiment were determined by western blot. (c) The transfection with pc-SelS plasmid and (d) transfection with SelS siRNA using in NO measurement were tested by western blot. (e) The pc-SelS plasmid transfection or (f) SelS siRNA transfection in eNOS mRNA testing was determined by RT-qPCR.
